# Historic range dynamics in Kaiser's mountain newt (*Neurergus kaiseri*): Insights from phylogeographic analyses and species distribution modeling

**DOI:** 10.1002/ece3.7595

**Published:** 2021-05-02

**Authors:** Somaye Vaissi

**Affiliations:** ^1^ Department of Biology Faculty of Science Razi University Kermanshah Iran

**Keywords:** amphibian, climate change, climate refugia, conservation, *Neurergus kaiseri*, Zagros mountain

## Abstract

Vulnerable Kaiser's mountain newt, *Neurergus kaiseri*, is endemic to highland streams, springs, and pools of the southwestern Zagros mountain, Iran. The present study aimed to use an integration of phylogeographical and species distribution modeling (SDM) approaches to provide new insights into the evolutionary history of the species throughout Quaternary climate oscillations. The phylogeographical analysis was followed by analyzing two mitochondrial DNA (mt‐DNA) markers including 127 control region (D‐loop) and 72 NADH dehydrogenase 2 (ND2) sequences from 15 populations in the entire species range that were obtained from GenBank. Potential recent and past distribution (the Last Glacial Maximum, LGM, 21 Kya and the Mid‐Holocene, 6 Kya) reconstructed by ensemble SDM using nine algorithms with CCSM4, MIROC‐ESM, and MPI‐ESM‐P models. *N*. *kaiseri* displayed two distinct lineages in the northern and southern regions that diverged in the Early‐Pleistocene. The demographics analysis showed signs of a slight increase in effective population size for both northern and southern populations in the Mid‐Pleistocene. Biogeography analysis showed that both vicariance and dispersal events played an important role in the formation of recent species distribution of *N. kaiseri*. Based on SDM projection onto paleoclimatic data, *N. kaiseri* displayed a scenario of past range expansion that followed by postglacial contraction. The models showed that the distribution range of the species may have shifted to a lower altitude during LGM while with amelioration of climatic during Mid‐Holocene to recent conditions caused the species to shift to the higher altitude. The findings of the current study support the hypothesis that the Zagros mountains​ may be acting as climatic refugia and play an important role in the protection of isolated populations during climate oscillations.

## Graphical abstract

Phylogeographical analysis followed by two mitochondrial DNA (mtDNA) marker and potential recent and past distribution (the Last Glacial Maximum, LGM, 21 Kya and the Mid‐Holocene, 6 Kya) reconstructed by ensemble species distribution modeling using nine algorithms with CCSM4, MIROC‐ESM, and MPI‐ESM‐P models. Biogeography analysis showed that both vicariance and dispersal events played an important role in the formation of recent species distribution of *N. kaiseri*. Based on SDM projection onto paleoclimatic data, *N. kaiseri* displayed a scenario of past range expansion that followed by post‐glacial contraction.

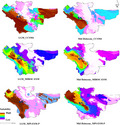

## 1 INTRODUCTION

In the Pleistocene (2.58 million to 11,700 years ago) especially at the Last Glacial Maximum (LGM; 23–19 Kya), glaciers covered about 30% of the Earth's surface (Head, 2019), creating a significant impact on the recent spatial distribution patterns and abundance of various animal and plant species as well as the genetic structure and demographic history of their populations (Arcones et al., 2021; Borràs & Cursach, 2021; Cornejo‐Romero et al., 2017; Ikeda et al., 2017; Pasquale et al., 2020). During this time, the distribution of temperate species often restricted into lower altitudes (or latitude) in the glacial refugia where climatic conditions were less extreme (Provan & Bennett, 2008). However, with the amelioration of climatic conditions in the Holocene (11,700 years ago to present time), the geographic range of species recolonized and expanded to higher altitudes (Bennett & Provan, 2008; Hampe & Petit, 2005). On other hand, the gene flow of individuals in the glacial refugia has approximately blocked leading to the allopatric divergence of populations (April et al., 2013; Canestrelli et al., 2012; Du et al., 2020). Nevertheless, the gene exchange during postglacial expansion could have obliterated the signs of historic isolation during glacial periods (Wang et al., 2015).

The Zagros mountains with an area of about 533,543 km^2^ formed by the collision of the Eurasian and Arabian plates during Miocene to Early‐Pliocene that has expanded in the western and southwestern Iranian plateau, ending at the Strait of Hormuz in Iran, northeastern Iraq, and southeastern Turkey (Agard et al., 2005). The sharp environmental gradient of the Zagros mountains at the conjunction of the Mesopotamian plain provides a remarkable region with high spatial and climatic variation, protecting various species with high taxonomic and genetic diversity (e.g., Afroosheh et al., 2019; Ghaedi et al., 2020; Kafash et al., 2020; Kazemi & Hosseinzadeh, 2020). The paleoecological and palynological evidence indicated during LGM, the Zagros mountains were characterized by a cooler and more arid climate compared to the Holocene (Djamali et al., 2012; Kehl, 2009). By some estimates, it has been shown that temperatures in the Zagros mountains were about 5°C lower than in the present time (Bobek, 1963). During this time, the lower altitudes of Zagros mountains were covered by the spread of steppe habitats and the higher altitudes by glaciers, whose remains are still visible in the highest mountains including the Zardeh Kuh (Preu, 1984), and possibly Kuh‐e Dinar in the central Zagros and Kuh‐i‐Jupar, Kuh‐i‐Lalezar and Kuh‐i‐Hezar Massifs in the southern Zagros (Kuhle, 2008). Also, there is some evidence of phylogeographic and paleoclimatic modeling that has highlighted Zagros mountains acting as climatic refugia for some species during glacial–interglacial cycles (Afroosheh et al., 2019; Ahmadzadeh et al., 2013; Fathinia et al., 2020; Malekoutian et al., 2020; Rajaei Sh et al., 2013).

The genus *Neurergus* involves four currently recognized species (*N. derjugini*, *N*. *kaiseri*, *N. crocatus,* and *N. strauchii*), which are specific for their small ranges restricted to mountainous areas in western Iran, northeastern Iraq, and southern Turkey (Hendrix et al., 2014). The Kaiser's mountain newt (*Neurergus kaiseri*, Schmidt 1952) is endemic to highland first‐order streams, springs, and pools constructed on karst springs (altitudinal range: 385–1,500 m) in southwestern Zagros mountain, Iran (Sharifi et al., 2013; Vaissi & Sharifi, 2019). Based on IUCN criteria *N. kaiseri* is considered a vulnerable species (IUCN; Red List criteria: B1ab (iii,v)). Moreover, *N*. *kaiseri* has been annexed to Appendix I of the Convention to the International Trade to Endangered Species (CITES), (Sharifi et al., 2009). The major threats to this species include degradation of habitats and fragmentation and diversion of water from highland streams to orchards and agricultural lands (Sharifi et al., 2009). Also, the disturbing impact of climate change, which has caused many springs and small streams to completely dry up, threatens the survival of amphibian populations (Sharifi et al., 2009). Prior studies on the population genetic structure and niche modeling of this species revealed two highly differentiated clades in the north and south of the Dez river (Farasat et al., 2016; Goudarzi et al., 2019). The future projection of distributions for this species indicates reduced spatial connectivity and continued habitat loss (Ashrafzadeh et al., 2019).

The present study traces the evolutionary history of Kaiser's mountain newt in southwestern Zagros mountain and test hypotheses concerning the response of species from this area to climatic oscillation during the Quaternary by the integration of the statistical phylogeographic analyses and species distribution modeling (SDM). Determining how past climate change has influenced the distribution and diversification of species can help us understand how anthropogenic climate change will impact their persistence (Forester et al., 2013). Therefore, this study may improve future conservation planning that could be specific to particular lineages of geographically restricted sections of a species range (D’Amen et al., 2013). For this purpose, data on two mitochondrial DNA (mt‐DNA) markers including NADH dehydrogenase 2 (ND2) and control region (D‐loop) were extracted from the GenBank (a) to investigate the biogeographical history and historical demographic of *N. kaiseri* in the entire species range and (b) to determine past range dynamics by reconstructing potential distributions for the climatic conditions of the LGM (21 Kya) and the Mid‐Holocene (6 Kya).

## 2 MATERIALS AND METHODS

### 2.1 Phylogeny and divergence time estimates

**FIGURE 1:**
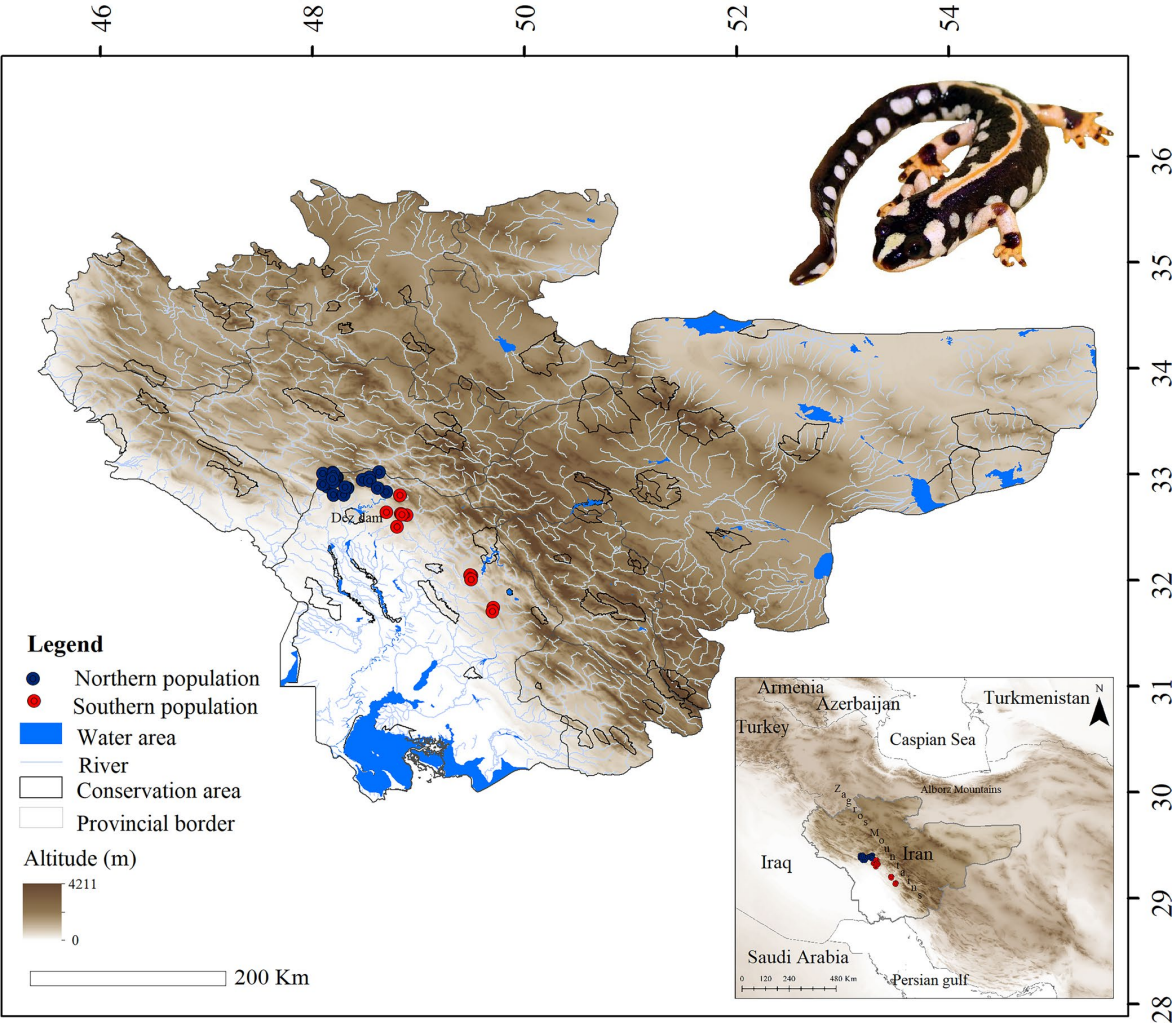
Study area. Blue circles represent the northern population, and red circles represent the southern population of the Kaiser's mountain newt, *Neurergus kaiseri* in the southwestern Zagros mountain, Iran

Data of two mitochondrial (mt‐DNA) genes including 127 control region (D‐loop: Farasat et al., 2016) and 72 NADH dehydrogenase 2 (ND2: Vaissi & Sharifi, 2021) sequences from 15 populations in the entire species range were obtained from GenBank (NCBI) and used for phylogenetic analysis (Figure 1). Details of the sequence data, outgroups, and their accession numbers are provided in Table S1.

Although, various phylogenetic studies have shown the existence of two northern and southern lineages in the *N. kaiseri* (Farasat et al., 2016; Goudarzi et al., 2019; Khoshnamvand et al., 2019; Vaissi & Sharifi, 2021). However, in this study based on ND2 and D‐loop sequences, Bayesian inference (BI) in MrBayes v 3.2.2 (Ronquist, Huelsenbeck, & Teslenko, 2011) was used for the construction of the phylogenetic tree. It should be noted phylogenetic tree constructed by haplotypes that were computed using the DnaSP v 5.10.01 (Rozas et al., 2010).

BEAST v 2.5 was used to estimate divergence times between different lineages of *N. kaiseri* based on combined mitochondrial ND2 and D‐loop genes (Bouckaert et al., 2019). Bayesian Markov Chain Monte Carlo (MCMC) was used in conjunction with the uncorrelated lognormal relaxed clock and the calibrated Yule (Heled & Drummond, 2012). Calibration was carried out based on the evolutionary rate of the ND2 gene by Weisrock et al. (2001), that is 0.64% per million years (Mya) per lineage and D‐loop gene by Steinfartz et al. (2000) that is 0.80% per Mya per lineage. The best fit model identified by Akaike information criterion was the GTR model for ND2 gene and HKY model for D‐loop gene using the jModelTest v 0.1.1 (Posada, 2008). This analysis runs based on 10 million generations with 1,000 generations for every sampling. Tracer v 1.6 (Rambaut et al., 2007) was used to check convergence and parameter estimates with ESS values >200. Generated trees to find the maximum credibility tree were obtained by TreeAnnotator v1.8.4 (Drummond & Rambaut, 2007). Finally, the tree generated and visualized using FigTree v 1.4.0 (Rambaut, 2012).

### 2.2 Demographic analysis

Demographic history was analyzed for the total, northern and southern population using both the ND2 and D‐loop genes. Arlequin v 3.5 (Excoffier & Lischer, 2010) was used to the demographic history of the species including Neutrality test analysis, that is, the Tajima's *D*, the Fu's *Fs*, the sum of squared deviation (SSD), Harpending's raggedness index (RAG), and mismatch distribution (MMD). Investigation of the variations in effective population size (*N*_e_) against time was constructed by Bayesian skyline plot (BSP). BEAST v 2.5 under the strict clock at the rate of 0.64% (for ND2) and 0.80% (for D‐loop) per Mya per lineage (Steinfartz et al., 2002; Weisrock et al., 2001) was used for BSP analysis. The analysis was run for 10 million generations with log parameters sampled every 1,000 generations. Tracer v 1.6 was used to estimate effective population size through time.

### 2.3 Biogeography analysis

Based on the previous studies (Farasat et al., 2016; Goudarzi et al., 2019; Khoshnamvand et al., 2019) and phylogenetic tree (Figure S1), the geographical range of *N. kaiseri* was divided into the northern and the southern distribution range. Two models of historical biogeography analysis were used to reconstruct the possible ancestral ranges including statistical dispersal‐vicariance analysis (S‐DIVA) and Bayesian binary MCMC (BBM) that implemented by Range Ancestral State in Phylogeny (RASP; Yu et al. (2015)). The tree obtained from the BEAST analysis from combining genes was used as the input file. The number of trees for RASP analysis was 10,001.

### 2.4 Occurrence and environmental data

The study area included two provinces containing the *N. kaiseri* (Lorestan and Khuzestan provinces) and seven neighboring provinces (Kohgiluyeh and Boyer‐Ahmad, Chaharmahal and Bakhtiari, Isfahan, Markazi, Hamedan, Kermanshah, and Ilam) in southwestern Iran (Figure 1). The occurrence points of Kaiser's mountain newt, *N. kaiseri,* were obtained from Vaissi and Sharifi (2019) totaling 38 unique records. The minimum distance between the occurrence points (Dodut spring and Moolik spring) was 280 m, and the maximum distance (Abliseneh and Dare Palangi) was 209 Km. The average migration distance in closely related species (*N. derjugini*) is about 49.19 ± 71.75 m (Afroosheh & Sharifi, 2014). But to be sure, all multiple records of sites within a minimum distance of 500 m are excluded to reduce the impacts of repetitive occurrences made at specific sites (Moolik spring and Choobeh). This selection process reduced occurrence records to 36 data points that were used for the distribution modeling approach.

Last Glacial Maximum (21 Kya), the Mid‐Holocene (6 Kya), and recent climatic data including 19 bioclimatic variables were downloaded from the WorldClim database which were 2.5 arc‐min resolution (https://www.worldclim.org). Climatic data for the LGM and Mid‐Holocene were derived from three atmospheric circulation models (ACM): CCSM4 (Community Climate System Model Version 4: see Gent et al. (2011)), MIROC‐ESM (Model for Interdisciplinary Research on Climate‐Earth system models: see Kawamiya et al. (2020)) and MPI‐ESM‐P (Max Planck Institute‐Earth System Model: see Giorgetta et al. (2013)). To exclude the highly correlated WorldClim bioclimatic variables, we computed the Pearson correlations among all bioclimatic variables and neglected those over *r* > .75. Finally, six bioclimatic variables were used to run the models include annual mean temperature (BIO1); mean diurnal range (mean of monthly (max temp ‐ min temp), (BIO2); temperature annual range (BIO5‐BIO6), (BIO7); annual precipitation, (BIO12); precipitation of driest quarter, (BIO17); and precipitation of warmest quarter (BIO18).

### 2.5 Species distribution modeling

Biomod2 package in R v 4.0.30 was used to ensemble SDM (Thuiller et al., 2016). Statistical methods all have disadvantages and advantages, so various statistical methods are often employed together to improve habitat suitability estimation (Elith et al., 2006; Friedman, 1991; Hastie et al., 1994; Leathwick et al., 2006; Zuur et al., 2010). Biomod2 not only offers such a platform for ensemble forecasting but also overcomes existing limitations of other software that are not able to fit and compare single‐algorithm models (Thuiller et al., 2009). For this propose, nine algorithms were run: three modern machine‐learning methods, generalized boosted models (GBM: Ridgeway, 1999), random forest (RF: Breiman, 2001), and artificial neural networks (ANN: Ripley, 1996); three regression methods, generalized linear models (GLM: McCullagh & Nelder, 1989), generalized additive models (GAM: Hastie & Tibshirani, 1990), and multivariate adaptive regression splines (MARS: Friedman, 1991); one enveloping method, surface range envelops (SRE: Busby, 1991); and two classification methods, flexible discriminant analysis (FDA: Hastie et al., 1994) and classification tree analysis (CTA: Breiman et al., 1984).

These models are based on the presence–absence algorithms, and since the absence records were not available, the pseudo‐absence records with a number similar to the records of presence were randomly generated for each model (Guisan et al., 2017). For each model, 70% of the data were randomly assigned for model calibration and 30% for the performance of the algorithms. To prevent bias from the splitting of the total records, each model algorithm was run 10 times (Ancillotto et al., 2020; Ashrafzadeh et al., 2019; Gilani et al., 2020; Guan et al., 2020; Zhang et al., 2021). True skill statistic (TSS: score >0.8), the area under the receiver operating characteristic curve (AUC: score >0.8), and Cohen's Kappa (KAPPA: score >0.8) used the predictive performance of each model (Guisan et al., 2017). ArcMap v 10.4.1 was used for all the spatial analyses.

## 3 RESULTS

### 3.1 Phylogeny and divergence time estimates

**FIGURE 2:**
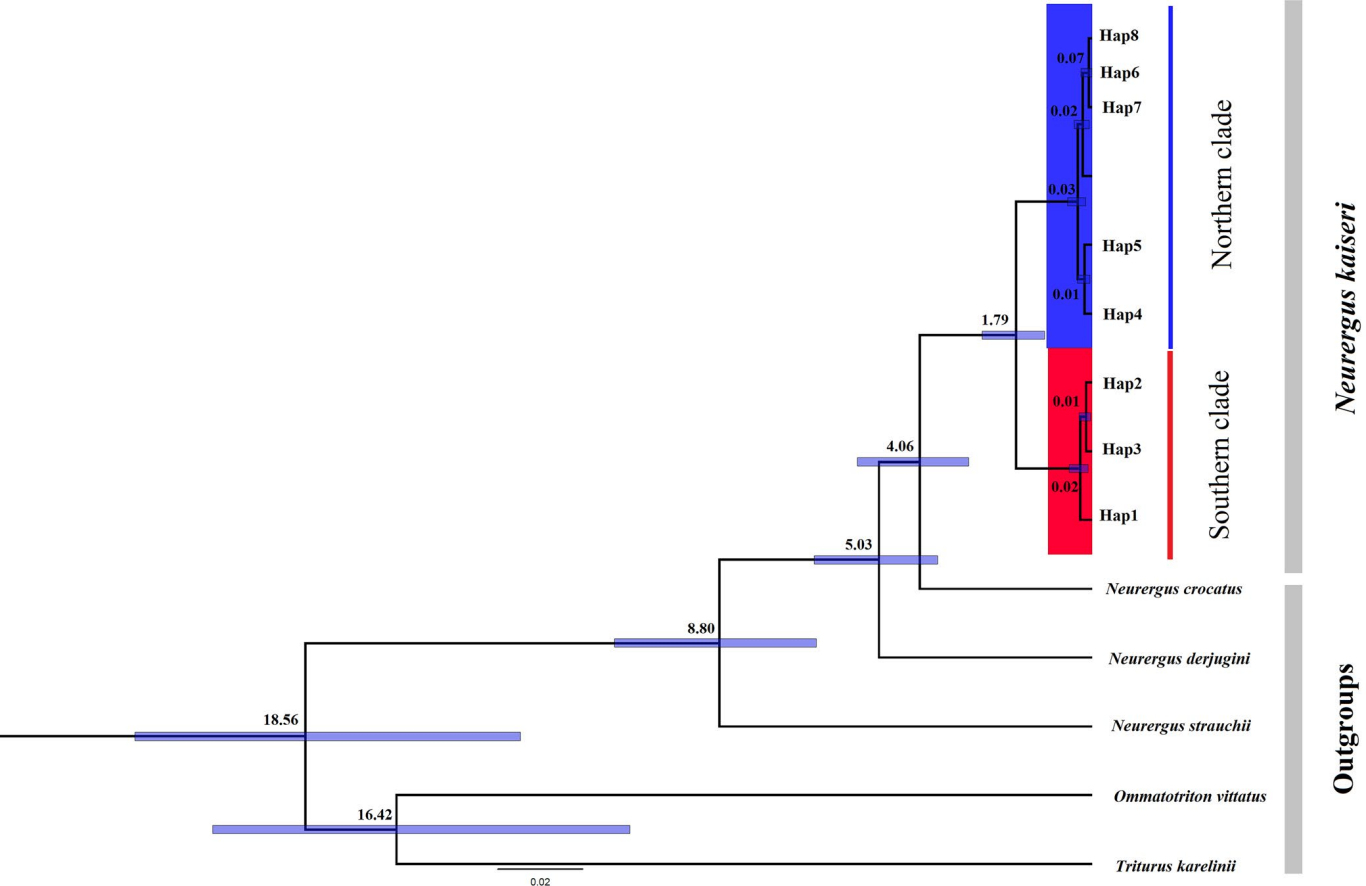
A calibration evolutionary time tree based on combining two mt‐DNA (ND2 and D‐loop) genes for Salamandridae including the genus *Neurergus*. Blue bars show 95% highest posterior density intervals of the estimated node ages; values indicated on branches are mean node ages (Mya)

Based on the combined ND2 and D‐loop sequences, BI trees showed that *N. kaiseri* haplotypes divided into two distinct clades in the northern and southern distribution range (Figure S1). Based on divergence time estimation, *N. derjugini* and *N. kaiseri* have diverged approximately 5.03 Mya (Figure 2). This separation for *N. kaiseri* and *N. crocatus* occurred approximately 4.06 Mya (Figure 2). Divergence times for southern from northern population fell within Early‐Pleistocene origin (95% HPD, approximately 1.79 Mya); (Figure 2).

### 3.2 Demographic analysis

**FIGURE 3:**
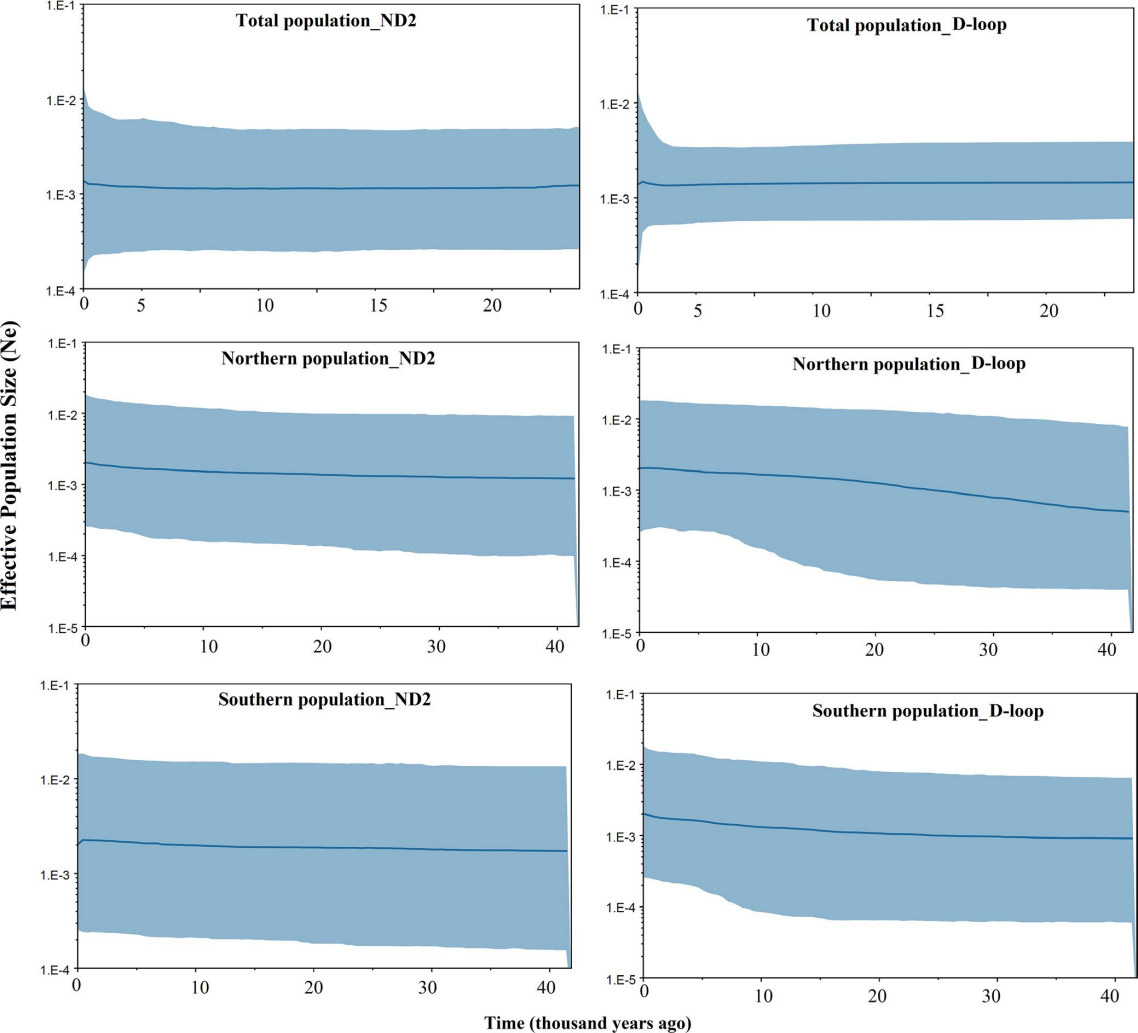
Bayesian skyline plot based on two mt‐DNA (ND2 and D‐loop) genes. The central line shows the median values of the population size (thousand years), and the blue area represents the 95% highest posterior density

Tajima's *D* and Fu's *Fs* values, the SSD, and RAG (Harpending's raggedness index) within *N. kaiseri* and its clades are shown in Table 1. The bimodal pattern, not significant SSD, and RAG in the entire species range may be suggesting constant population size (Table 1, Figure S2). However, bimodal peaks may also reveal the presence of two distinct lineages in the northern and southern distribution range. Based on the ND2 sequences, the MMD diagrams for the northern and southern populations of *N. kaiseri* showed a bimodal distribution, while based on D‐loop sequences, a unimodal pattern was observed for the northern and southern populations of *N. kaiseri* which may indicate signs of a recent demographic expansion (Figure S2). Bayesian skyline plots based on ND2 (tau = 26.32) and D‐loop (tau = 8.93) sequences indicated a constant in effective population in total populations (Figure 3). However, a slight increase in effective population size was observed for the southern (ND2 = 30 and D‐loop = 23 Kya) and northern (ND2 = 22 and D‐loop = 38 Kya) populations especially in D‐loop sequences (Figure 3).

**TABLE 1:** Tajima's *D* and Fu's *Fs* values, the sum of squared deviation (SSD), and RAG (Harpending's raggedness index) within *Neurergus kaiseri* and its clades

		Tajima's *D* (*p*)	Fu's *Fs* (*p*)	SSD (*p*)	Harpending's Raggedness index (*p*)
Total	ND2	2.45 (.99)	17.13 (.99)	0.10 (.13)	0.08 (.17)
	D‐loop	2.32 (.98)	5.64 (.93)	0.14 (.07)	0.19 (.13)
Northern population	ND2	0.43 (.39)	1.47 (.78)	0.06 (.20)	0.18 (.17)
	D‐loop	−0.59 (.32)	−0.97 (.25)	0.002 (.33)	0.17 (.38)
Southern population	ND2	0.98 (.81)	2.52 (.89)	0.43 (.000)	0.19 (.99)
	D‐loop	−0.67 (.25)	−0.75 (.23)	0.005 (.39)	0.27 (.59)

### 3.3 Biogeography analysis

**FIGURE 4:**
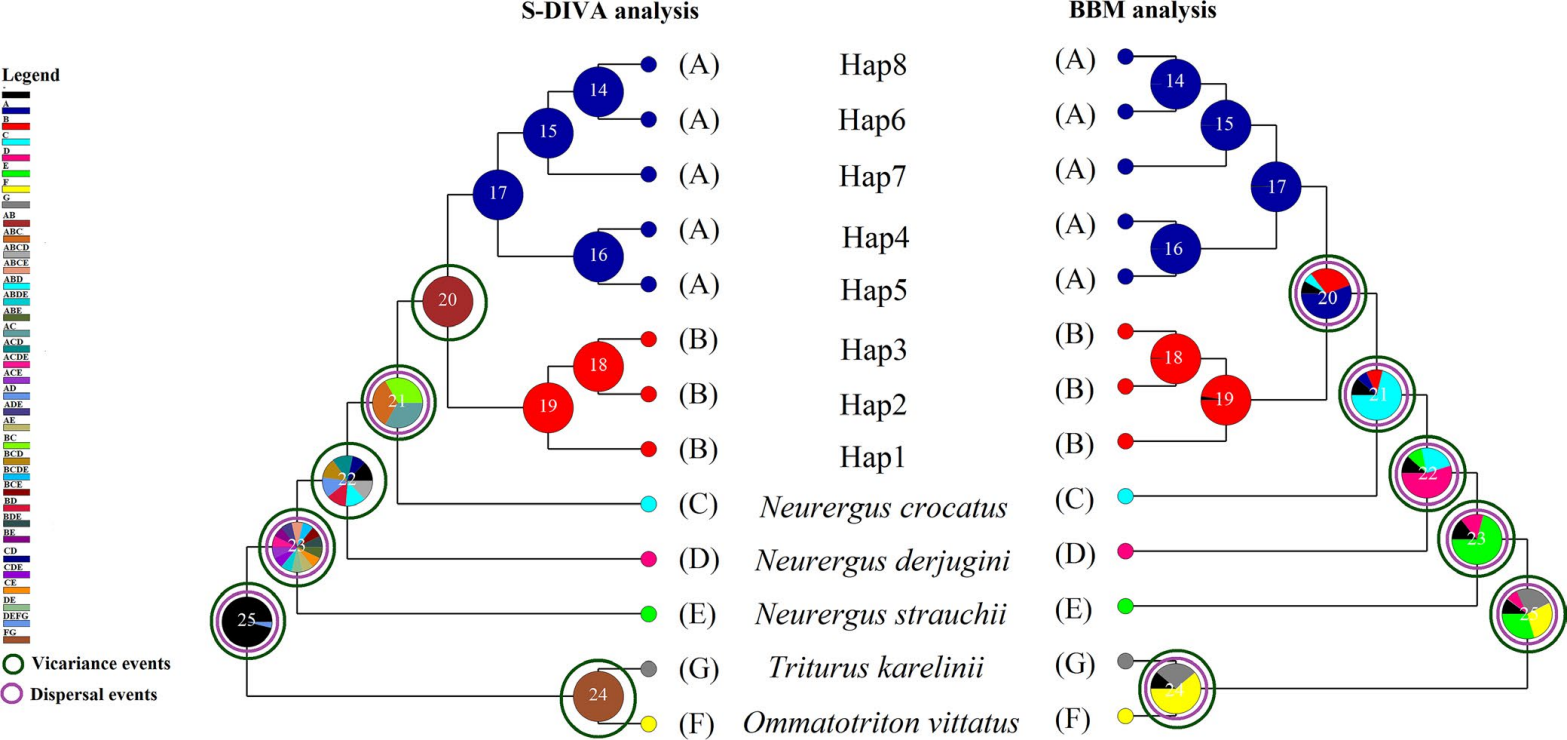
The biogeographic analysis of *Neurergus* with S‐DIVA and BBM analysis based on combining two mt‐DNA (ND2 and D‐loop) genes. (A: red nodes) *Neurergus kaiseri*: northern distribution, southwestern Iran. (B: blue nodes) *N. kaiseri*: southern distribution, southwestern Iran. (C: phosphor nodes) *Neurergus crocatus*: northeastern Iraq, southeastern Turkey and northwestern Iran. (D: pink nodes) *Neurergus derjugini*: western Iran and northeastern Iraq. (E: light green nodes) *Neurergus strauchii*: southeastern Turkey. (G: gray nodes) *Triturus karelinii*: Crimea, Caucasus and south of the Caspian Sea. (F: yellow nodes) *Ommatotriton vittatus*: Armenia, Iraq, Israel, Jordan, Lebanon, Syria, and Turkey

The statistical dispersal‐vicariance analysis (S‐DIVA) and Bayesian binary MCMC (BBM) for the reconstruction of the possible ancestral ranges of *N. kaiseri* are indicated in Figure 4. Based on S‐DIVA analysis, the ancestors of the *N. kaiseri* (node 20) were presented in the entire species range, which was divided into northern and southern populations by the vicariance event (Figure 4). However, based on the BBM analysis, the ancestors of southern populations were presented in the south of the distribution range and the ancestors of northern populations were presented in the north of the distribution range (Figure 4). According to BBM reconstruction, both dispersal and vicariance events have been implicated in the recent formation of species distribution (Figure 4).

### 3.4 Species distribution modeling

In most of the models, the high predictive capacity of true skill statistic (TSS), ROC curve (AUC), and Cohen's Kappa (KAPPA) indicate the high sensitivity (false positive rate) and specificity (true positive rate); (Table 2). Uncorrelated predictors and mean (±*SD*) of their contributions (%) in nine *N. kaiseri* distribution models from high to low are the precipitation of warmest quarter (BIO18: 40.62% ± 11.07), annual precipitation (BIO12: 13.71% ± 2.38), temperature annual range (BIO7: 13.65 ± 6.94), mean diurnal range (BIO2: 12.32% ± 2.14), annual mean temperature (BIO1: 11.92 ± 5.88), and precipitation of driest quarter (BIO17: 7.78 ± 0.53).

**TABLE 2:** True skill statistic (TSS), ROC curve (AUC), and Cohen's Kappa (KAPPA) of nine used algorithms projecting *Neurergus kaiseri* distribution in southwestern Zagros mountain, Iran

Scenarios		GLM	GBM	RF	GAM	CTA	ANN	SRE	FDA	MARS
CCSM4	KAPPA	1	0.97	1	1	0.97	1	0.87	0.50	1
	TSS	1	0.97	1	1	0.97	1	0.86	0.50	1
	AUC	1	1	1	1	0.99	1	0.93	0.76	1
MIROC‐ESM	KAPPA	1	0.97	1	1	0.95	1	0.87	0.79	0.92
	TSS	1	0.97	1	1	0.95	1	0.86	0.79	0.92
	AUC	1	1	1	1	0.97	1	0.93	0.87	1
MPI‐ESM‐P	KAPPA	1	1	1	1	0.95	1	0.84	0.34	0.89
	TSS	1	1	1	1	0.95	1	0.83	0.34	0.90
	AUC	1	1	1	1	0.97	1	0.92	0.67	0.96

**FIGURE 5:**
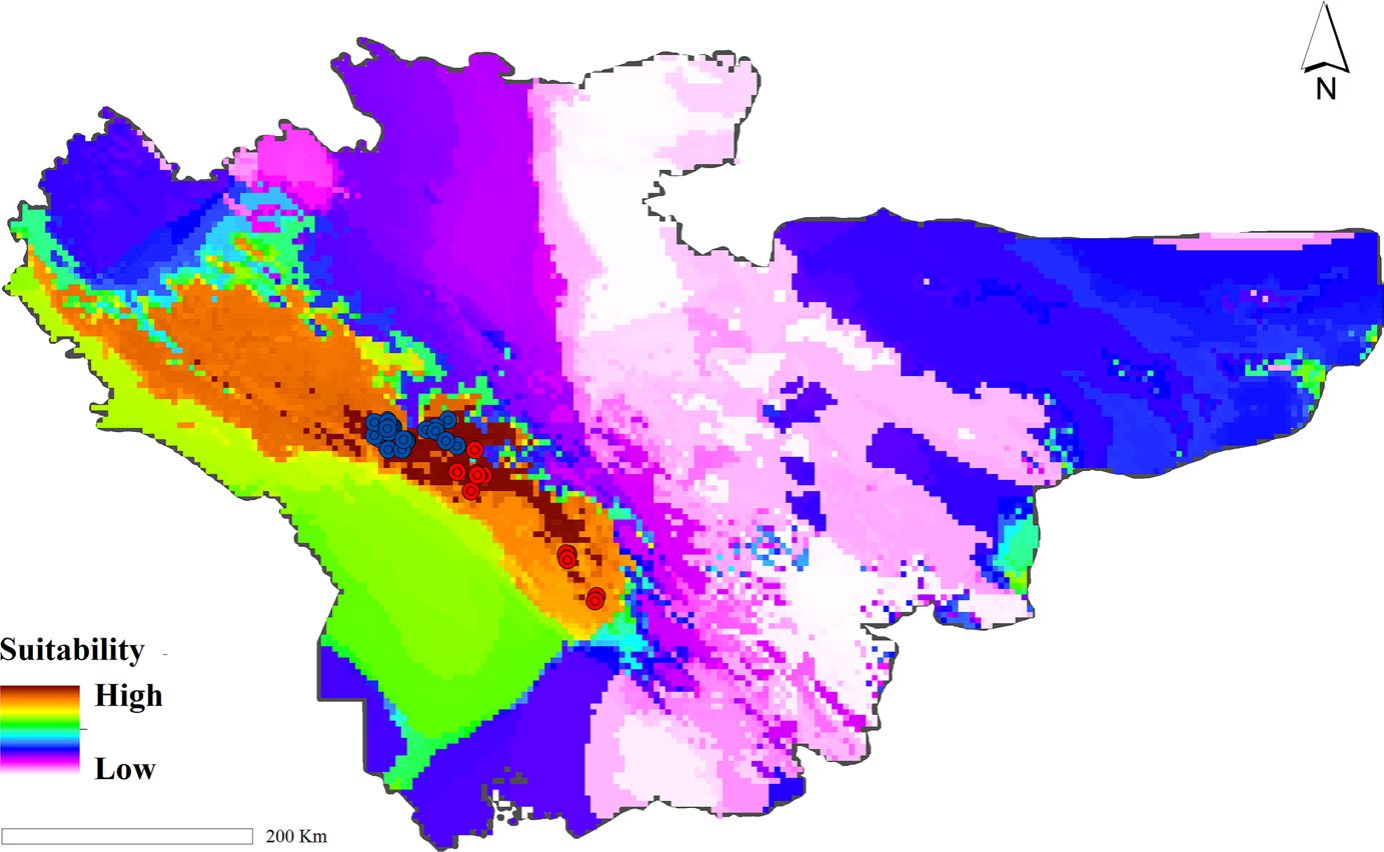
Potential distribution range of the Kaiser's mountain newt, *Neurergus kaiseri* in the southwestern Zagros mountain under recent climate condition

**FIGURE 6:**
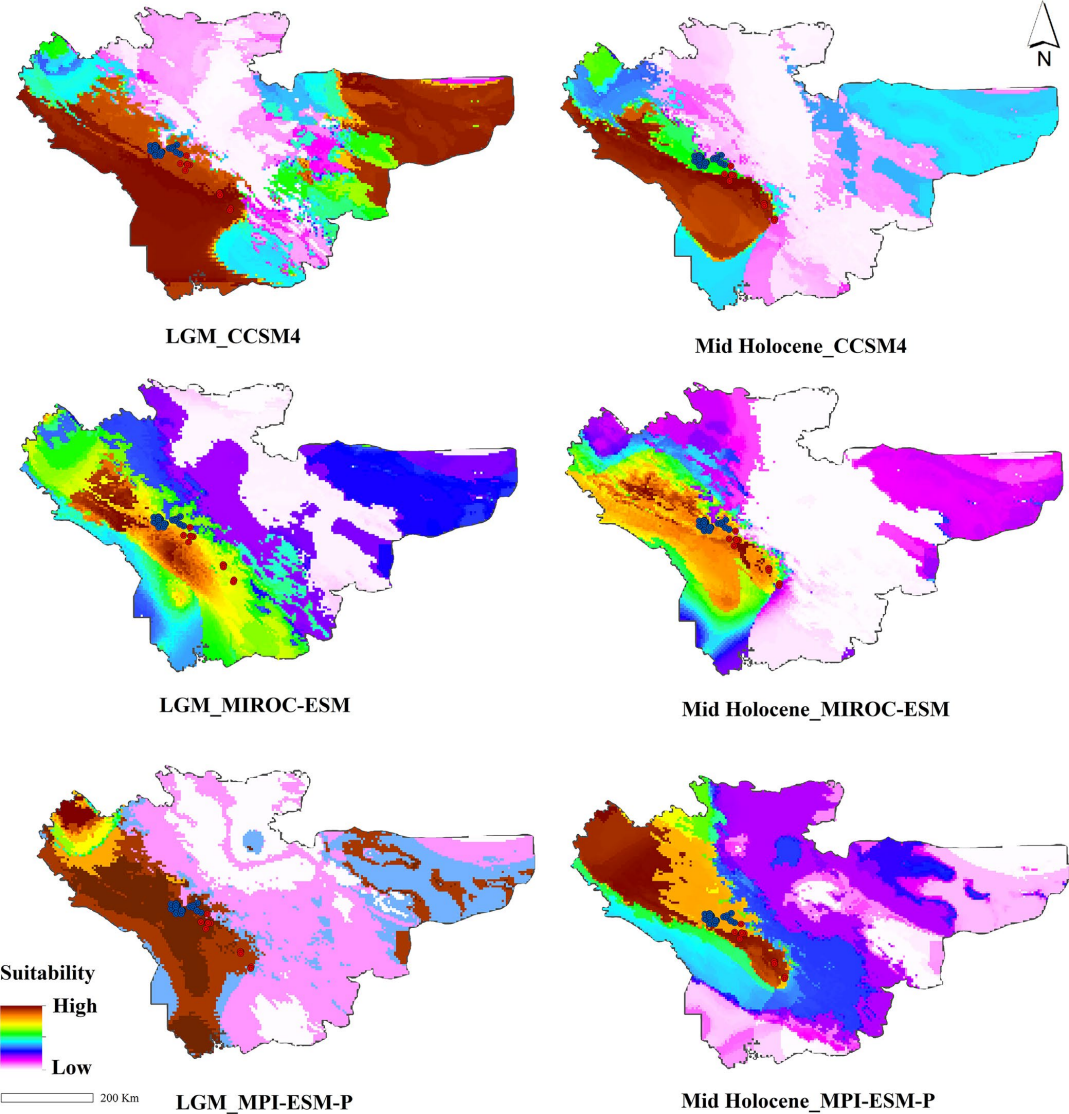
Potential distribution range of the Kaiser's mountain newt, *Neurergus kaiseri* in the southwestern Zagros mountain under Last Glacial Maximum (LGM, 21 Kya) and the Mid‐Holocene (6 Kya) climate condition

The potential distribution range of the *N. kaiseri* for the recent climatic conditions is shown in Figure 5. Last Glacial Maximum, LGM (21 Kya), and Mid‐Holocene (6 Kya) distribution range based on CCSM4, MIROC‐ESM, and MPI‐ESM‐P models are shown in Figure 6. According to all three models, during the LGM, *N. kaiseri* had a wider and more suitable habitat than the Mid‐Holocene and recent climate conditions in the northern and southern part of the distribution range (Figure 6). During the LGM, the distribution range of *N. kaiseri* is more distributed at lower altitudes, while in the Mid‐Holocene to the recent climatic conditions, the range of species is more shifted toward higher altitudes (Figure 6).

## 4 DISCUSSION

Several studies have focused on the genetic structure and phylogeny of the vulnerable Kaiser's mountain newt, *N. kaiseri* (Farasat et al., 2016; Goudarzi et al., 2019; Khoshnamvandet al., 2019). However, the present study, using phylogeographical analysis and SDM, for the first time, investigated the impact of historical processes on shaping the genetic diversity and contemporary distribution of *N. kaiseri*, throughout Quaternary climate oscillations.

The *Neurergus* and its sister taxa, *Triturus*, evolved from a common ancestor distributed in Europe and the Mediterranean region. Later, some members of this ancestral taxon dispersed to the south and distributed the Zagros and surrounding areas (Zhang et al., 2008). Afterward, the vicariance event resulting from Zagros mountains orogeny and dispersal has played an important role in radiation, isolation, speciation, and subsequent evolution of the *Neurergus* (Steinfartz et al., 2002). In fact, over time, with the rise of the Zagros mountains (9–10 Mya) and the emergence of high and narrow mountain valleys, the ancestors of *Neurergus* were divided into several isolated populations (allopatric species), which led to the blockage of gene flow and formation of complete reproduction isolation (Steinfartz et al., 2002). As a result, four species were formed, including *N*. *strauchii* in southeastern Turkey; *N. crocatus* in northeastern Iraq, southeastern Turkey and northwestern Iran; *N. derjugini* in western Iran and northeastern Iraq, and *N. kaiseri* in southwestern Iran (Steinfartz et al., 2002). The results of the biogeographical analysis in this study also highlight that both dispersal and vicariance events have participated in the evolution of different species of the genus *Neurergus*, although the role of vicariance seems to be more prominent (Figure 4). Based on two mt‐DNA markers, *N. kaiseri* diverged from *N. crocatus* approximately 4.06 Mya in the Early‐Pliocene. *Neurergus kaiseri* diverged from *N. derjugini* approximately 5.03 Mya in the Late‐Miocene. The divergence of the *N. strauchii* from the *N. crocatus*, *N. derjugini,* and *N. kaiseri* has estimated approximately 8.80 Mya in the Late‐Miocene.

The presence of two clades across the northern and southern distribution range of *N. kaiseri* has been displayed by various mitochondrial and nuclear markers (Farasat et al., 2016; Goudarzi et al., 2019; Khoshnamvand et al., 2019; Vaissi & Sharifi, 2021). In this study, the presence of two lineages was also confirmed using a bimodal curve in MMD analysis (Figure S2). The evaluation of Bayesian skyline plots displays that insignificant expansion in *N. kaiseri* population began at about 22 (ND2) and 38 (D‐loop) Kya in northern and 30 (ND2) and 23 (D‐loop) Kya in the southern population during Quaternary glaciations (Figure 3). The divergence time between the two lineages of *N. kaiseri* was estimated at approximately 1.79 Mya in the Early‐Pleistocene (Figure 2). Both S‐DIVA and BBM analysis from biogeographical history inferred that the vicariance events played an important role in the formation of recent species distribution of *N. kaiseri* (Figure 4). The BBM analysis displayed that dispersal also played a role in this divergence. According to the result, this dispersal has occurred in both northern and southern lineages. Based on S‐DIVA analysis, the ancestors of the *N. kaiseri* were present in the entire distribution range (Figure 4). According to BBM analysis, the ancestors of the southern lineages were present in the south of the distribution range and the ancestors of the northern lineages were present in the north of the distribution range (Figure 4). Either way, this result agrees with a recent study by Goudarzi et al. (2019) that showed that the divergence between the two lineages is due to the Dez river, which formed about ~3–3.5 Mya in the Late‐Pliocene (Oberlander, 1965). The present study also suggests that gradual speciation from the Late‐Pliocene to the Early‐Pleistocene is more likely.

The biogeographical analysis provided in the present study seems to be more supported by SDM. Based on SDM projection onto paleoclimatic data in all three models (CCSM4, MIROC‐ESM, and MPI‐ESM‐P), *N. kaiseri* displayed a scenario of past range expansion that followed by postglacial contraction (Figures 5 and 6). According to the result, both precipitation and temperature have a significant impact on the distribution of *N. kaiseri*. It seems that climatic conditions for *N. kaiseri* during the LGM were favorable in an extensive area than recent climate condition (Figure 6). In the LGM, the species may have occupied areas in lower altitudes situated westward of the current distribution range (Figure 6). The gradual rise of temperature in the south‐Zagros mountains after the LGM and in the Mid‐Holocene likely forced *N. kaiseri* to shift their distribution upward to higher altitudes (Figure 6); (Yousefi et al., 2015). This disagrees with the typical reaction of many amphibian species in the western Palearctic, which retracted their ranges to smaller geographical areas known as glacial refugia (Alexandrino et al., 2000). However, the cold‐adapted species commonly displayed this pattern of range expansion during LGM and contraction the range during postglacial warming (Afroosheh et al., 2019; Kearns et al., 2014; Teixeira et al., 2018). For example, closely related mountain newts, the Yellow‐spotted mountain newt, *N. derjugini*, also displayed the same pattern of LGM expansion that followed by a recent contraction (Afroosheh et al., 2019). Finally, this study suggests that the southern parts of the Zagros mountains can act as a climate refugia and is a valuable area for biodiversity conservation (Ashcroft, 2010; Gavin et al., 2014; Hampe et al., 2013).

## CONFLICT OF INTERESTS

None declared.

## AUTHOR CONTRIBUTIONS

**Somaye Vaissi:** Conceptualization (lead); Data curation (lead); Formal analysis (lead); Funding acquisition (lead); Investigation (lead); Methodology (lead); Project administration (lead); Resources (lead); Software (lead); Supervision (lead); Validation (lead); Visualization (lead); Writing‐original draft (lead); Writing‐review & editing (lead).

## ETHICS APPROVAL AND CONSENT TO PARTICIPATE

This study complied with the appropriate institutional, national, and international guidelines.

## Supporting information

Supplementary Material

## Data Availability

NADH dehydrogenase 2 (ND2) and control region (D‐loop) haplotypes used in this study were extracted from the NCBI Nucleotide Database. Details of accession numbers and their references are provided in Table S1. Last Glacial Maximum (LGM, 21 Kya), the Mid‐Holocene (6 Kya) and recent climatic data downloaded from the WorldClim database (https://www.worldclim.org).
