# Supplementary material for: Historic range dynamics in Kaiser's mountain newt (Neurergus kaiseri): Insights from phylogeographic analyses and species distribution modeling
Source: Ecol Evol. 2021 May 2;11(12):7622–33. doi: 10.1002/ece3.7595 (PMC8216884; doi:10.1002/ece3.7595)
Supplement: Supplementary file 1 — Supplementary Material [file ECE3-11-7622-s001.doc]

**TABLE S1** The data set used in this study for phylogeographic analyses and ensemble species distribution modelling. The information includes locality, geographical coordinates, number of analyzed individuals, haplotype number, origin, and the accession numbers for two mt-DNA (ND2 and D-loop) genes

|  | | | | **ND2** | | | **D-loop** | | |
| --- | --- | --- | --- | --- | --- | --- | --- | --- | --- |
| **No.** | **Locality #** | **Latitude/ Longitude** | **Altitude (m.a.s.l.)** | **Number of analyzed individuals** | **Haplotype number** | **Accession number** | **Number of analyzed individuals** | **Haplotype number** | **Accession number** |
| 1 | Abjdan | 32°02'/49°30' | 841 | 5 | Hap1 | MW655886 † | 12 | Hap1,  Hap2 | KP748175 ‡  KP748176 ‡ |
| 2 | Bolhasan | 32°36'/48°50' | 925 | 5 | Hap2 | MW655887 † | 5 | Hap1 | KP748175 ‡ |
| 3 | Deje-e Mohammad Ali Khan | 32°36'/48° 49' | 1094 | 5 | Hap2 | MW655887 † | 12 | Hap1 | KP748175 ‡ |
| 4 | Labsefid | 32°33'/48°49' | 852 | 5 | Hap2 | MW655887 † | 5 | Hap1 | MW680844 † |
| 5 | Emamzadeh Haft Tanan | 32°36'/48°53' | 848 | 2 | Hap2 | MW655887 † | 2 | Hap1 | MW680844 † |
| 6 | Shevi (Talezang) | 32°47'/48°49' | 813 | 5 | Hap3 | MW655888 † | 10 | Hap1,  Hap2,  Hap3 | KP748175 ‡  KP748176 ‡  KP748181 ‡ |
| 7 | Shahzadeh Ahmad (Bozorgab waterfall) | 32°56'/48°28' | 1332 | 5 | Hap4,  Hap5 | MW655889 †  MW655890 † | 12 | Hap5,  Hap6 | KP748177 ‡  KP748180 ‡ |
| 8 | Cheshmeh Sila | 32°52'/48°18' | 1070 | 5 | Hap6 | MW655891 † | 5 | Hap4 | KP748179 ‡ |
| 9 | Daregol | 32°57'/48°12' | 1227 | 5 | Hap7 | MW655892 † | 11 | Hap4 | KP748179 ‡ |
| 10 | Hajibarikab | 32°51'/48°20' | 1004 | 5 | Hap6,  Hap8 | MW655891 †  MW655893 † | 11 | Hap4,  Hap5 | KP748179 ‡  KP748177 ‡ |
| 11 | Kerki | 32°58'/48°11' | 936 | 5 | Hap7 | MW655892 † | 5 | Hap4 | MW680846 † |
| 12 | Kerser | 32°58'/48°12' | 995 | 5 | Hap7 | MW655892 † | 11 | Hap4,  Hap5 | KP748179 ‡  KP748177 ‡ |
| 13 | Pifeh | 32° 57'/48°32' | 1381 | 5 | Hap4,  Hap7 | MW655889 †  MW655892 † | 9 | Hap4,  Hap7 | KP748178 ‡  KP748180 ‡ |
| 14 | Shahzadeh Ahmad (Dodut spring) | 32°55'/48°32' | 1280 | 5 | Hap4 | MW655889 † | 5 | Hap4 | KP748179 ‡ |
| 15 | Tafo | 32°57'/48°14' | 1387 | 5 | Hap7 | MW655892 † | 12 | Hap4  Hap5 | KP748179 ‡  KP748177 ‡ |
| 16 | Sargach Ezeh | 31°44'/49°42' | 1015 | - | - | - | - | - | - |
| 17 | Shaikhon | 32°51'/48°19' | 960 | - | - | - | - | - | - |
| 18 | Vojenab | 33°00'/48°37' | 930 | - | - | - | - | - | - |
| 19 | Tove | 32°49'/48°41' | 990 | - | - | - | - | - | - |
| 20 | Shahbazan | 32°47'/48°42' | 1200 | - | - | - | - | - | - |
| 21 | Dareeh dioni | 32°38'/48°41' | - | - | - | - | - | - | - |
| 22 | Shovalander | 32°02'/49°29' | - | - | - | - | - | - | - |
| 23 | Mazoo | 32°51'/48°37' |  | - | - | - | - | - | - |
| 24 | Darkhorma | 32°59'/48°07' | - | - | - | - | - | - | - |
| 25 | Voroun Nargeseh | 32°52'/48°08' | 647 | - | - | - | - | - | - |
| 26 | Mordestan | 33°00'/48°11' | 928 | - | - | - | - | - | - |
| 27 | Koolchap | 33°55'/48°12' | 847 | - | - | - | - | - | - |
| 28 | Doolshali | 32°54'/47°54' | 768 | - | - | - | - | - | - |
| 29 | Abkesh | 32°54'/48°06' | 908 | - | - | - | - | - | - |
| 30 | Abliseneh | 33°00'/48°06' | 1081 | - | - | - | - | - | - |
| 31 | Doolnesar | 33°00'/48°12' | 1313 | - | - | - | - | - | - |
| 32 | Dare palangi | 31°42'/49°42' | 857 | - | - | - | - | - | - |
| 33 | Keremb | 32°00'/49°30' | 488 | - | - | - | - | - | - |
| 34 | Abzaleh | 32°54'/48°12' | 1257 | - | - | - | - | - | - |
| 35 | Chenar mongreh | 32°38'/48°12' | 957 | - | - | - | - | - | - |
| 36 | Cheshmeh ziad | 32°51'/48°20' | 990 | - | - | - | - | - | - |
| 37 | *Neurergus crocatus* | - | - | - | - | DQ517788 * | - | - | - |
| 38 | *Neurergus strauchii* | - | - | - | - | DQ517791 * | - | - | EU880321 ∞ |
| 39 | *Neurergus derjugini* | - | - | - | - | DQ517790 * | - | - | KP748182 ‡ |
| 40 | *Triturus karelinii* | - | - | - | - | DQ517837 * | - | - | HQ697277 ∞ |
| 41 | *Ommatotriton ophryticus* | - | - | - | - | DQ517844 * | - | - | EU880338 ∞ |
| *: Origin: Weisrock et al., 2006; ∞ Origin: Zhang et al., 2008; ‡ Origin: Farasat et al., (2016); # Origin: Vaissi and Sharifi, (2019); † Origin: Vaissi and Sharifi, (2021) | | | | | | | | | |

**
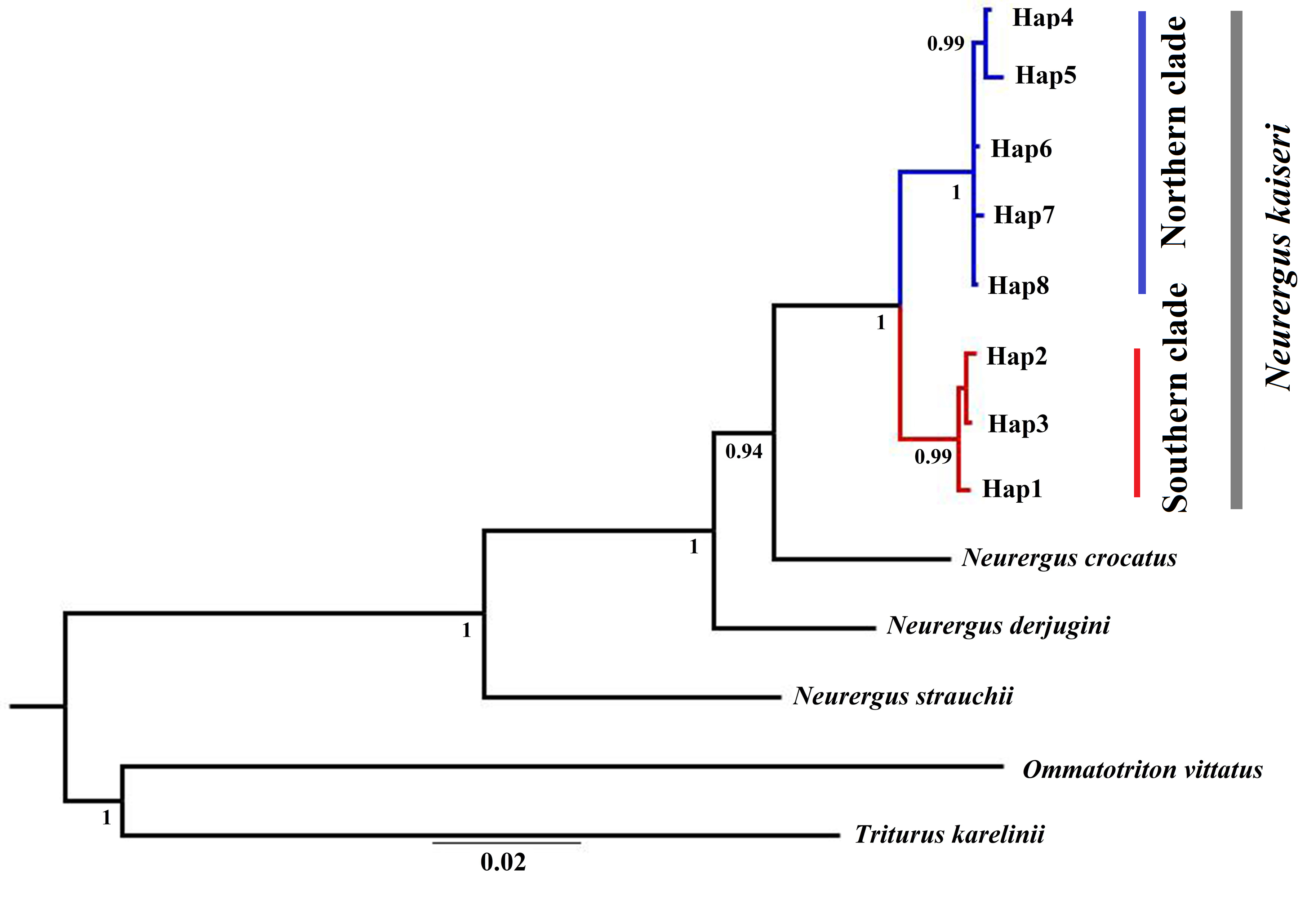
**

**FIGURE S1** MrBayes tree of *Neurergus kaiseri* based on combining two mt-DNA (ND2 and D-loop) genes. Numbers below branches indicate the posterior probabilities of the nodes in the Bayesian inference analysis

**
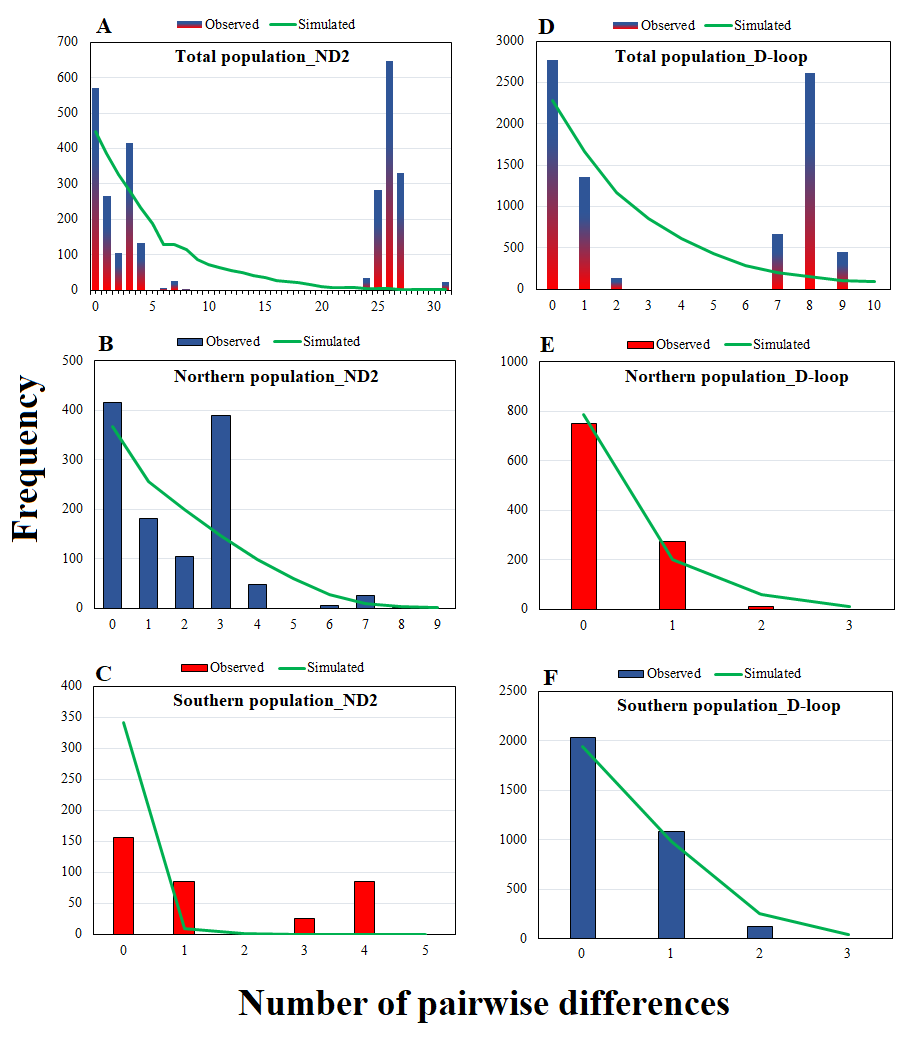
**

**FIGURE S2** Mismatch distributions of expected frequencies compared to the observed frequencies based on two mt-DNA (ND2 and D-loop) genes in the total, northern and southern populations of the Kaiser's mountain newt, *Neurergus kaiseri*
